# Integrated transcriptomic and metabolomic data reveal the cold stress responses molecular mechanisms of two coconut varieties

**DOI:** 10.3389/fpls.2024.1353352

**Published:** 2024-04-16

**Authors:** Jing Li, Fangyuan Wang, Md. Abu Sayed, XiaoJun Shen, Lixia Zhou, Xiaomei Liu, Xiwei Sun, Shuangyan Chen, Yi Wu, Lilan Lu, Shufang Gong, Amjad Iqbal, Yaodong Yang

**Affiliations:** ^1^ Coconut Research Institute, Chinese Academy of Tropical Agricultural Sciences/Hainan Key Laboratory of Tropical Oil Crops Biology, Wenchang, Hainan, China; ^2^ School of Tropical Crops, Yunnan Agricultural University, Kunming, Yunnan, China; ^3^ Department of Food Science & Technology, Abdul Wali Khan University Mardan, Mardan, Pakistan

**Keywords:** coconut, varieties, cold stress, transcriptome, metabolome

## Abstract

Among tropical fruit trees, coconut holds significant edible and economic importance. The natural growth of coconuts faces a challenge in the form of low temperatures, which is a crucial factor among adverse environmental stresses impacting their geographical distribution. Hence, it is essential to enhance our comprehension of the molecular mechanisms through which cold stress influences various coconut varieties. We employed analyses of leaf growth morphology and physiological traits to examine how coconuts respond to low temperatures over 2-hour, 8-hour, 2-day, and 7-day intervals. Additionally, we performed transcriptome and metabolome analyses to identify the molecular and physiological shifts in two coconut varieties displaying distinct sensitivities to the cold stress. As the length of cold stress extended, there was a prominent escalation within the soluble protein (SP), proline (Pro) concentrations, the activity of peroxidase (POD) and superoxide dismutase (SOD) in the leaves. Contrariwise, the activity of glutathione peroxidase (GSH) underwent a substantial reduction during this period. The widespread analysis of metabolome and transcriptome disclosed a nexus of genes and metabolites intricately cold stress were chiefly involved in pathways centered around amino acid, flavonoid, carbohydrate and lipid metabolism. We perceived several stress-responsive metabolites, such as flavonoids, carbohydrates, lipids, and amino acids, which unveiled considerably, lower in the genotype subtle to cold stress. Furthermore, we uncovered pivotal genes in the amino acid biosynthesis, antioxidant system and flavonoid biosynthesis pathway that presented down-regulation in coconut varieties sensitive to cold stress. This study broadly enriches our contemporary perception of the molecular machinery that contributes to altering levels of cold stress tolerance amid coconut genotypes. It also unlocks several unique prospects for exploration in the areas of breeding or engineering, aiming to identifying tolerant and/or sensitive coconut varieties encompassing multi-omics layers in response to cold stress conditions.

## Introduction

Coconut (*Cocos nucifera L.*), a tropical lush evergreen plant, native to Southeast Asia and the Pacific Islands belongs to the Palmae family (Xiao et al., 2017; Yousefi et al., 2023). It is mainly scattered in the subtropical and tropical zones of the world. Coconut holds considerable value both in terms of edibility and economic significance amongst tropical fruit trees. Nutrient-rich coconuts can be savored both in their fresh state and crafted into a wholesome beverage (Kumar et al., 2021; Shen et al., 2024). In addition, mature coconut meat remains an imperative primary resource for extracting coconut oil. Besides, processed products derived from coconut leaves, roots, stems, and shells also represent substantial economic value (Roopan, 2016).

Low temperature poses a critical challenge, impeding the natural growth of coconuts and serving as one of the detrimental abiotic stresses that affect their topographical distribution (Yang et al., 2018). The optimal temperature for coconut growth is 27-32°C, with yearly average temperature of 29°C (Beveridge et al., 2022). Low temperature not only inflicts severe damage on low-temperature sensitive coconut varieties at seedlings stage, but also leads to fruit drop, uneven accumulation of coconut flesh and decreased quality of coconut water in mature plants. Consequently, there is an urgent need to understand the mechanism and cultivate coconut varieties that are tolerant to cold stress.

Recent studies have shown that plants have adapted intricate operations enabling them to sense external cues and portray adaptive behaviors through suitable structural adjustments and anatomical alteration (Zheng et al., 2021; Liu et al., 2022). Under frigid conditions, the plant cellular membrane shifts from a fluid crystalline to a solid gel phase (Chinnusamy et al., 2010). Interestingly, substantial quantities of reactive oxygen species (ROS) accumulate in plants after enduring a cold environment (Apel and Hirt, 2004). The presence of ROS leads to disruptions in cellular dynamics, causing oxidative injury (Dat et al., 2000). The level of cold tolerance varies among different genotypes, a characteristic linked to the generation of ROS, activities of antioxidant enzymes and antioxidant system (Islam et al., 2023). The primary ROS scavengers in plants are peroxidase (POD), ascorbate peroxidase (APX), catalase (CAT), glutathione peroxidase (GPX), and superoxide dismutase (SOD). Through the collaboration of these enzymes and antioxidants like ascorbic acid and glutathione, cells have efficient mechanisms to neutralize O_2_^-^ and H_2_O_2_ (Romero-Puertas et al., 2006; Dorigan de Matos Furlanetto et al., 2019). As of now, extensive research has been conducted on the involvement of GPXs in plant abiotic stress responses, encompassing *Arabidopsis*, chrysanthemum, tomato, and tobacco (Zhang et al., 2018; Gul et al., 2022; Yang et al., 2022).

There is a consensus that cold treatment triggers the expression and/or performance of many cellular mechanisms. Namely, sugars and amino acids are frequently acknowledged to be linked to the cold response. They are indispensable not only for the generation of active proteins but also as antecedents for a comprehensive range of biomolecules with multifaceted functions in plant responses to low temperature (Laura et al., 2010). Under cold stress, plants accumulate carbohydrates and amino acids, serving as osmotic protection agents. To survive under low temperatures, plants need to generate more energy carriers for the synthesis of amino acids, lipids, membrane elements, and other molecules. This action augments cell membrane flexibility and facilitates restructuring (Yoon et al., 2017). The amendment in innate GABA and stress-inducing agents trigger associations amongst amino acid biosynthesis, photosynthesis, and carbon and nitrogen metabolism. These deviations may aid in improving the cold resilience in tobacco (Xu et al., 2020) and tea plants (Zhu et al., 2019). Scientific inquests have admitted that improved amounts of flavonoids, like quercetin, kaempferol, and anthocyanins develop in tea plants during cold stress act as a defense against oxidative injuries (Zheng et al., 2016).

Integrated transcriptomics and metabolomics approaches are gaining prevalence to uncover molecular dynamics of abiotic stress resilience in various crops owing to genetical, morphological, and physiological data (Maruyama et al., 2014; Jiang et al., 2023; Li Q. et al., 2023). In spite of this, there have been limited studies evaluating coconuts under cold stress by analyzing metabolomics and transcriptomics data. Therefore, we performed a comparative analysis of the low-temperature stress response in two coconut varieties, identifying meaningful contrasts in their metabolic profiles and gene expression patterns. These distinctions encompassed core genes within routes linked with ROS tolerance, amino acids, and flavonoids biosynthesis. This suggests that these routes play a pivotal role in determining the varying degrees of cold stress tolerance between two coconuts varieties. Our results suggest that coconut varieties sensitive to cold stress exhibit reduced expression in flavonoids and amino acid biosynthesis, leading to their heightened susceptibility to cold stress. These results offer a promising starting point for comprehending the cold stress tolerance of coconuts it’s important to note that the underlying mechanisms are intricate and demand further in-depth study. Gaining a deeper insight into the molecular mechanisms governing coconuts at cold stress has the potential to facilitate the breeding of cold-tolerant coconut cultivars.

## Materials and methods

### Plant materials and treatments

Six-month-old seedlings of Hainan Tall coconut (HT) and Green Dwarf coconut (GD) cultivars used in this study were sourced from the nursery of the Coconut Research Institute, Chinese Academy of Tropical Agricultural Sciences in Wenchang, Hainan, P. R. China. In the experiment, seedlings grown at 25°C were designated as the control group (CK). In addition, low-temperature treatment groups were established at 8°C for 2 hours (2h), 8 hours (8h), 2 days (2d), and 7 days (7d). To ensure the representativeness of the experimental results, 45 healthy seedlings were selected for each variety, with 9 seedlings assigned for treatment at each designated time point. At each time point, the processed samples included three biological replicates. Each replicate was a mix of functional leaves from three seedlings. The seedlings were cultivated in a growth room for one week. For the control treatment, seedlings from both varieties were raised in controlled settings with 16 hours of light and 8 hours of darkness, and the temperature was kept at 25°C. After 1 week, all coconut seedlings, except those designated for the control group, were transferred to a controlled greenhouse for cold treatment at 8°C, with the same light-dark cycle. After producing a functional leaf, each seedling was harvested at its designated processing time points (CK, 2h, 8h, 2d, 7d). Afterwards, the leaf veins were removed from the harvested leaves and cut them into small pieces to ensure a consistent mix of leaf tissues from different plants. The cut leaf samples were quickly frozen in liquid nitrogen and stored in a refrigerator at -80°C for subsequent RNA extraction, transcriptome sequencing, and metabolomic analysis.

### Physiological index measurements

The proline content, glutathione peroxidase (GSH), soluble proline contents and the activity of catalase (CAT) in the samples were assessed with assay kits from Suzhou Grace Biotechnology Co., Ltd, Jiangsu, China. The activity of peroxidase (POD) and superoxide dismutase (SOD) were assessed with assay kits from Solarbio Science & Technology Co., Ltd, Beijing, China. Specifically, 0.1 g of fresh sample was ground and utilized for extraction, and the levels of soluble protein (SP), GSH, proline (Pro), catalase (CAT), SOD and POD were measured spectrophotometrically (UV2600, SHIMADZU, Japan) following the manufacturer’s instructions. All these processes were independently and simultaneously replicated three times.

### RNA extraction and RNA-Seq analysis

Tissue samples were taken from the functional leaves of two different varieties of coconut seedlings. The nine individual coconut seedlings were selected from each treatment. For each sample, three functional leaves from the seedlings subjected to the same treatment were cut and combined, forming a biological sample. This process was repeated three times to create biological replicates for each time period. The RNA extraction method followed the protocol outlined by Iqbal et al. (2019). The quality of the extracted RNA, including degradation and contamination, was evaluated using 1% agarose gel. Meanwhile, the coherence of the RNA was ascertained by Agilent 2100 Bioanalyzer (Agilent Technologies, CA, USA), and employed a Nanodrop Spectrophotometer (IMPLEN, CA, USA) for concentration assessment. Sequencing of the samples was done on the BGISEQ-MGI2000 platform instrument at BGI Genomics (Wuhan 430073, China), with three biological replicates for each sample. Following the sequencing process, clean reads were attained by filtering out those holding adapters, having more than 5% unknown bases and exhibiting low quality (bases with a quality score of ≤ 10 accounting for >20% of the sequence). Application of SOAPnuke (v1.4.0) facilitated the filtering of raw data (Chen et al., 2018), which involved removing reads with adapter contamination, unknown base ratios surpassing 5%, accompanied by inferior-quality base ratios exceeding 20%. Filtered sequences were subsequently retained in FASTQ format and the data were aligned to the reference genome utilizing HISAT (v2.1.0) (Kim et al., 2015), and then matched with the assembled unique genes exercising Bowtie2 (v2.2.5) (Langmead and Salzberg, 2012). RSEM (v1.2.8) was employed to estimate the degree of gene expression (Li and Dewey, 2011). The assembled Unigene sequences were annotated using functional databases such as KEGG and GO, and transcription factors were predicted. Differential gene analysis within groups was conducted using DESeq with the conditions of Fold Change greater than or equal to 2 and Adjusted P-value less than or equal to 0.001 (Wang et al., 2010).

### Metabolomics analysis by UPLC-MS/MS

Sample preparation for metabolomics and the subsequent data analysis were undertaken by BGI Genomics (Wuhan, 430070, China). The methods for sample extraction and determination are similar to those of Li et al. (2024), with some methods adjusted accordingly. In LC-MS analysis, take 20 uL of each sample mix it with QC samples to assess the reproducibility and stability of the LC-MS analysis. The experiment employed the Waters UPLC I-Class Plus (Waters, USA). The gradient conditions were set as follows: a linear increase from 5% to 95% B over 0.0-2.0 min, maintaining 95% B from 2.0-22.0 min, and a wash with 95% B over 27.1-30 min. Data preprocessing such as normalization and correction are carried out using the methods of Di Guida et al. (2016) and Dunn et al. (2011) for reference.

### Quantitative real-time PCR analysis

To validate the differentially expressed genes (DEGs) identified from transcriptome analysis, seven genes were randomly selected for a qPCR assay: BGI_novel_G001122 (phosphoglycerate kinase), COCNU_01G013540 (indole-3-glycerol phosphate synthase), COCNU_03G002950 (glutamine synthetase), COCNU_contig69087894G000010 (phosphoribosyl-ATP), COCNU_09G009610 (glutathione reductase (NADPH), COCNU_14G010360 (flavonol synthase) and COCNU_13G004330 (flavonol synthase). The CnACT gene used as an internal control as described previously (Xia et al., 2014). The primers used for qPCR analysis is provided in **Supplementary Table 2**.

RNA integrity and purity were scrutinized visually by agarose gel electrophoresis. The RNA abundance was quantified with a NanoDrop 2000 spectrophotometer (Thermo Fisher Scientific, Waltham, MA, USA). In the reverse transcription process, 1 μg of RNA was employed exhausting the MightyScript first-strand cDNA synthesis kit in accordance to the manufacturer’s instructions. Quantitative real-time PCR reactions were executed on a QuantStudio™ 6 Flex machine (Applied Biosystems, CA, USA) using the PowerUp™ SYBR™ Green Master Mix (Manufactured for Thermo Fisher Scientific Product, USA) following the provided instructions. The forward and reverse primers were developed exploiting Primer Premier 5 software, adhering to specific criteria, including melting temperatures within the range of 55-60°C, primer length between 19-22 bp, GC content ranging from 50-60%, and an amplicon size of 80-200 bp. To prevent amplification of non-target gDNA, the primers were designed to encompass intronic regions. Each primer’s properties were evaluated using the PCR Primer Stats software. The qPCR reactions were carried out with a total reaction mixture volume of 15 μL, following the amplification at 95°C for 5 s, 55°C for 15 s, and 68°C for 20 s. The melting stage involved heating from 60°C to 95°C for 20 min. Each experiment was conducted with biological and technical triplicates. The shift in expression level for each sample was computed by CT value normalization relative to a reference gene, exploiting the 2^^-ΔΔCt^ approach (Livak and Schmittgen, 2001).

### Data analysis

SPSS 16.0 for Windows and SAS software (SAS Inc., Cary, NC, USA) were utilized for data analysis. The mean ± standard deviations (P=0.05) were reported to summarize the experimental findings with a sample size of 3 (n=3). Correlation coefficients were calculated based on the mean values.

## Results

### Physiological response of coconut varieties against cold stress

**Figure 1:**
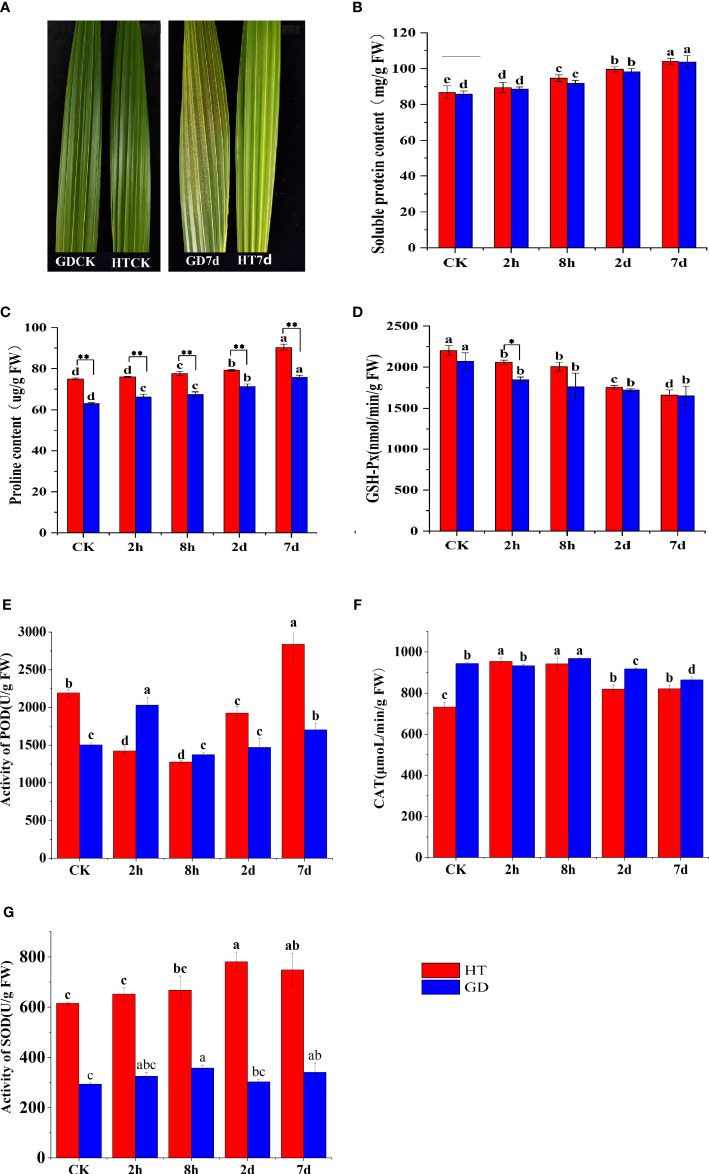
Different physiological responses in functional leaves of different coconut varieties treated with low temperature for 7 days Changes in HT and GD functional leaves before and after 7 days of low-temperature treatment **(A)**. The SP content **(B)**, Pro content **(C)**, GSH-Px activity **(D)**, POD activity **(E)**, CAT activity **(F)** and SOD activity **(G)** of coconuts under low temperature stress for CK, 2h, 8h, 2, and 7 days. The error bar represents SD of the mean of the three biological replicates. Different lower case letters (a–e) above the bars indicates significant differences between genes expression at different time point (p <0.05). * asterisks represent significant differences between the two varieties determined by Student’s t-test (*p < 0.05; **p < 0.01).

The results revealed that low temperature treatment directly affected the growth of coconut seedlings (**Figure 1A**). After 7 days of low-temperature treatment, noticeable yellow spots appeared on the functional leaves of GD coconut seedlings, while the functional leaves of HT retained their normal green appearance. Under cold stress, there was an increase in both soluble protein and proline contents. The proline content in two coconut varieties showed a rising trend with the prolonged duration of low-temperature treatment. Remarkably, the proline content in HT was higher than that in GD, and a highly significant difference was observed between the two groups. In comparison to the control, following 7 days of low-temperature treatment, the soluble protein content was increased by 21.9% for HT and 19.6% for GD. Likewise, after the same treatment duration, the proline content of HT and GD increased by 20.5% and 20.4%, respectively. (**Figures 1B, C**). Conversely, the glutathione peroxidase (GSH) levels in two coconut varieties exhibited a decreasing trend with the prolonged duration of low-temperature treatment. The GSH activity was higher in HT than in GD (**Figure 1D**). Additionally, there was no significant difference in the activity of CAT between HT and GD when exposed to 8°C (**Figure 1F**). However, the POD activity (**Figure 1E**) and the SOD activity (**Figure 1G**) were significantly higher in HT than GD. These results confirmed that HT was more cold-tolerant than GD.

### Global analysis of the metabolomic response

**Figure 2:**
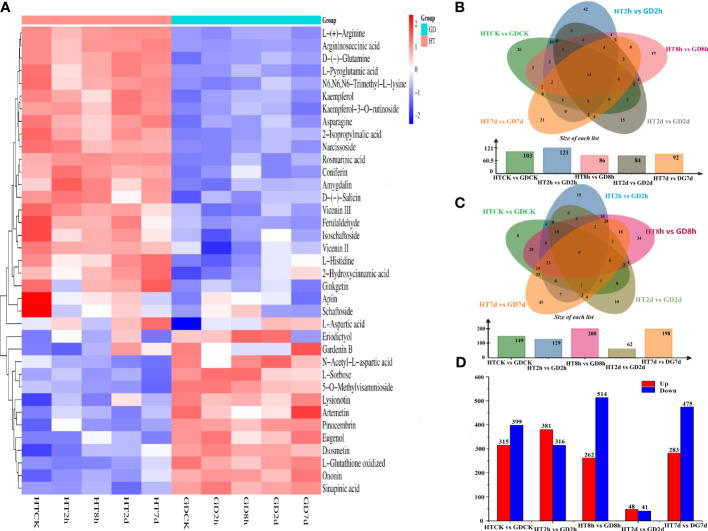
Metabolomic analysis of functional leaves of different coconut varieties under low-temperature treatment (n = 3). **(A)** Heatmap map analysis of DEMs for different samples under low-temperature treatment. The color code indicates the accumulation level of metabolites. The Venn plot shows the number of DEMs that are upregulated **(B)** and downregulated **(C)**. **(D)** The number of DEMs between five treatment cycles.

To gain a comprehensive understanding of the chemical changes in the functional leaves of two coconut varieties during cold stress, non-targeted metabolomics was employed. This involved utilizing UPLC-MS/MS for identifying shifts in metabolites during the course of five treatment periods. After averaging the metabolic data from three duplicate samples, the data were transformed by taking the log10 and represented as a heatmap. A total of 440 annotations from the KEGG database were categorized into 12 classifications. These classifications encompassed metabolism of nucleotides, carbohydrates, amino acids, lipids, terpenoids and polyketides, cofactors and vitamins, biosynthesis of other secondary metabolites, xenobiotics biodegradation and chemical structure transformation maps and so on. Among them, the generation of supplementary secondary metabolites accounted for the highest proportion, reaching 25%. Amino acid metabolism ranked second, reaching 15%, and the metabolism of terpenoids and polyketides was third, representing 14% (**Supplementary Figure S1**). A comprehensive identification process uncovered 1424 metabolites in total. These metabolites were classified into 40 categories, encompassing amino acids, carbohydrates, fatty acids, flavonoids, vitamins, nucleotides, terpenoids, polyketides, phenylpropanoids, nucleic acids, purines, steroids, benzene and derivatives, phenols, organic acids, cofactors, and amines and so on. Among these, carbohydrates; amino acids, peptides, and analogues; benzene and derivatives; terpenoids; flavonoids and lipids constituted 5.13%, 6.67%, 6.81%, 10.88%, 14.61%, and 17.56%, respectively (**Supplementary Figure S2**). The cluster analysis clearly divided the ten groups of samples into four distinct clusters (**Figure 2A**), suggesting significant shifts in the metabolite spectrum of coconut seedlings induced by cold stress. With the exception of L-Aspartic acid, various amino acid metabolites including L-(+)-Arginine, D-(-)-Glutamine, L-Pyroglutamic acid and aspartic acid were found to be more enriched in HT than in GD (**Figure 2A**).

The Venn diagram illustrated that fourteen (14) types of consistently upregulated DEMs (e.g., Arginine biosynthesis, Monoterpenoid biosynthesis and Lysine degradation and so on) were shared across all groups (**Figure 2B**), whereas 4 DEMs that were concurrently identified as downregulated (**Figure 2C**). Among the 14 co-upregulated metabolites, three were associated with amino acid metabolism. By comparing the common metabolites within each group, it was observed that across the five comparisons, only a limited set of metabolites were concurrently up- or down-regulated under the cold stress. This suggests a time-dependent regulation in coconut seedlings in response to the duration of cold treated. Among the five comparative groups, the number of upregulated metabolites was 43, whereas downregulated was 15 in HT2h vs GD2h. Conversely, in HT7d vs GD7d, the number of upregulated metabolites was 21 and the downregulated was 46. This pattern suggests that coconut seedlings activated a cold response mechanism in the face of cold stress.

The count of DEMs exhibiting upregulation and downregulation in HTCK-vs-GDCK, HT2h-vs-GD2h, HT8h-vs-GD8h, HT2d-vs-GD2d and HT7d-vs-GD7d were 315 and 399, 381 and 316, 262 and 514, 48 and 41, 283 and 475, respectively (**Figure 2D**). As indicated by our metabolomics data, in the HT2d vs GD2d comparison, the sum of upregulated and downregulated differential metabolites was the lowest among the five comparative groups. This observation suggests that the metabolic activity in coconut leaves might have experienced a decline after two days of cold stress, leading to a reduced number of detected differential metabolites.

### Differential transcriptomics in cold-stressed resistant and sensitive coconut varieties

To elucidate the distinct molecular responses of the two coconut varieties to low temperatures, we conducted a comparative analysis of transcriptomic data at 0h, 2h, 8h, 2d and 7d, for both resistant and susceptible coconut. The Illumina sequencing platform was used to collect raw and clean reads from 30 samples. In our analysis, normalization of gene expression levels was achieved through the use of the fragments per kilobase of transcript per million mapped reads (FPKM) approach. After sequencing quality control, a total of 192.8 GB of clean data was acquired from 30 samples. These samples included three biological replicates across five treatment periods for each of the two experimental samples. In each sample, the percentage of Q30 bases consistently stayed above 91.1%, indicating high sequencing quality. According to the statistics, he proficiency of comparing reads within each sample and the reference genome extended from 89.44% to 92.73% (**Supplementary Table 1**). The numbers of DEGs in coconut with more than a 2-fold change in expression (P < 0.05) were calculated for the comparisons HTCK-vs-GDCK, HT2h-vs-GD2h, HT8h-vs-GD8h, HT2d-vs-GD2d and HT7d-vs-GD7d. The total was HTCK-vs-GDCK: 2579 DEGs (1245 upregulated, 1334 downregulated), HT2h-vs-GD2h: 3155 DEGs (1666 upregulated, 1489 downregulated), HT8h-vs-GD8h: 2527 DEGs (1269 upregulated, 1258 downregulated), HT2d-vs-GD2d: 732 DEGs (346 upregulated, 386 downregulated), HT7d-vs-GD7d: 2869 DEGs (1691 upregulated, 1178 downregulated) (**Supplementary Figure S3**). The numbers of upregulated and downregulated DEGs in HT2h-vs-GD2h were considerably larger than those observed after 7days of treatment. This suggests that the most substantial transcription and translation processes may be activated or inhibited during the initial response to cold stress treatment. These results indicated that different durations of cold stress treatment lead to distinct gene expression profiles. The number of upregulated and downregulated genes in HT2d vs GD2d is the lowest among the five comparative groups, which may be attributed to the reduced metabolic activity in leaves after two days of cold stress, resulting in a decrease in differential genes (**Supplementary Figure S3**).

### Annotation and enrichment analysis of DEGs using KEGG pathways

**Figure 3:**
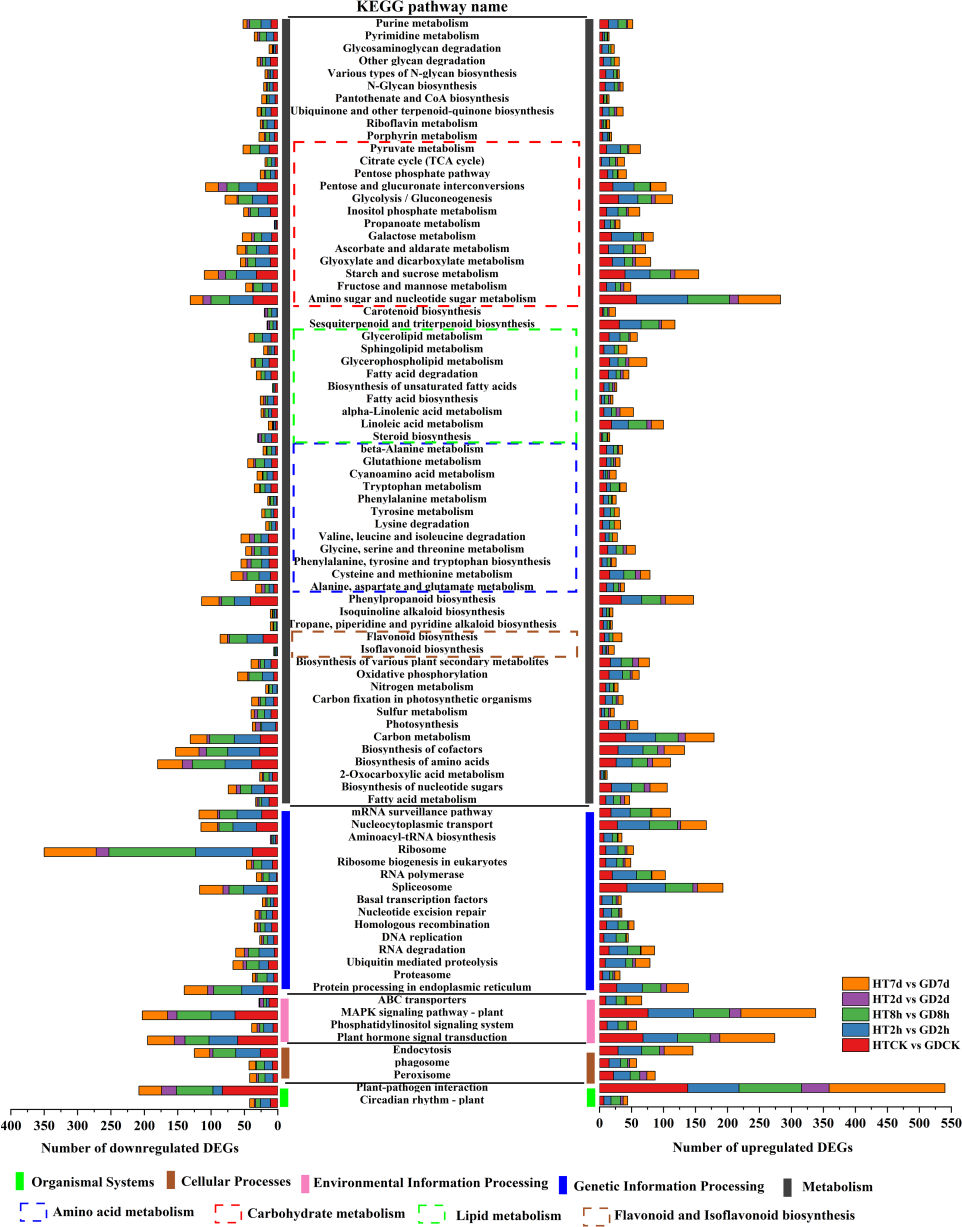
KEGG pathways enriched in all comparisons of HTCK vs GDCK, HT2h vs GD2h, HT8h vs GD8h, HT2d vs GD2d, HT7d vs GD7d and classification of DEGs.

To facilitate a comprehensive comparison of gene functions in the functional leaves of two coconut varieties under low-temperature treatment, enrichment analysis based on KEGG pathways was conducted. Annotated DEGs across diverse pathways to understand molecular processes associated with the observed gene expression changes. The pathways were categorized into 5 distinct groups, and investigated the DEG count within KEGG’s first and second-tier categories. Venn analysis revealed enrichment in a total of 36 pathways across all comparisons (**Supplementary Figure S4**). **Figure 3** displays the allocation of these pathways to 6 first-level KEGG classifications. In carbohydrate metabolism, the number of upregulated genes outweighed the number of downregulated genes in all five comparative groups. However, the metabolic pathways of flavonoids and isoflavones exhibited an opposite pattern (**Figure 3**).

The first-level class of “metabolism” was linked to the largest count of pathways and DEGs. Within this category, “global and overview maps” was particularly notable, being related to the largest number of both up- and down-regulated DEGs. In the context of “energy metabolism,” more upregulated DEGs were connected with both the CK and the cold stress time of 7days. Meanwhile, in the categories of “carbohydrate metabolism”, “lipid metabolism”, “Amino sugar and nucleotide sugar metabolism” and “global and overview maps”, more upregulated DEGs were observed. On the other hand, in “amino acid metabolism” and “Ribosome”, more downregulated DEGs were noted across all the cold stress periods. At the first-tier classification of “genetic information processing”, the subclass “replication and repair” were predominantly marked by downregulated DEGs at cold stress periods of 2h or 8h, shifting to upregulated DEGs during the cold stress periods of 2d and 7d. “Transcription” exhibited a prevalence of downregulated DEGs throughout the entire period of cold stress. At 8h, the number of downregulated genes was 191, whereas the upregulated base was 105, with a difference of 86. In “Folding, sorting and degradation”, downregulated DEGs dominated during the early cold stress of CK or 2h, transitioned to upregulated DEGs in the middle period of 8h and 2d, and ultimately returned to downregulated DEGs at culmination of 7d. Remarkably, regarding the first-tier classification of “environmental information processing”, “plant hormone signal transduction” emerged as predominantly featured with the greatest number of DEGs. Both “plant hormone signal transduction” and “MAPK signaling pathway - plant” were marked by an extended count of downregulated DEGs throughout the entire period of cold stress (**Figure 3**).

### Correlation between differentially expressed genes and metabolites

**Figure 4:**
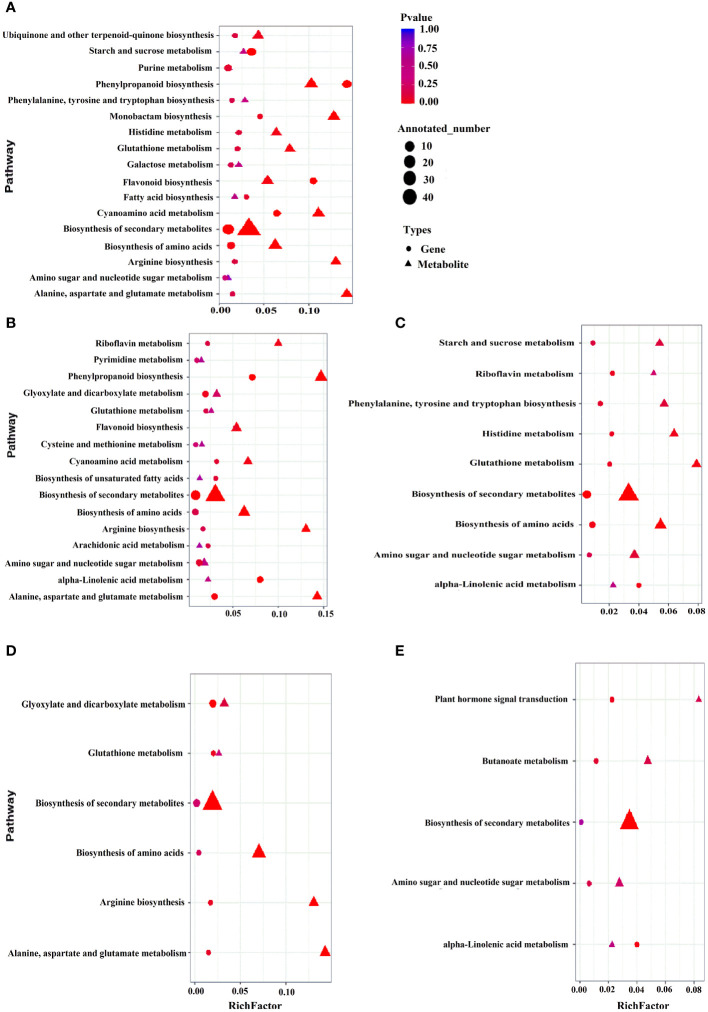
Correlation analysis of differentially expressed genes and differential metabolites in different KEGG pathways. HT_CK_-vs-GD_CK_, HT_2h_-vs-GD_2h_, HT_8h_-vs-GD_8h_, HT_2d_-vs-GD_2d_ and HT_7d_-vs-GD_7d_ **(A–E)**. The abscissa is the ratio of the differential metabolites or differentially expressed genes enriched in the pathway to the number of metabolites or genes annotated in the pathway, and the ordinate is the common enrichment of metabolome-transcriptome. The count is the number of metabolites or genes enriched in the pathway.

To comprehend the regulatory networks governing the two coconut varieties addressing cold stress, by performing correlation analysis with DEGs obtained from transcriptomics and metabolomics analysis. The data was subsequently utilized to create a comprehensive metabolic map (**Figure 4**). The map illustrated pathways related to the biosynthesis of secondary metabolites, amino acids, flavonoids and phenylpropanoid were enriched under control conditions (HTCK-vs-GDCK) and during early stage of cold stress (HT2h-vs-GD2h) (**Figures 4A, B**). Under cold stress treatment, there was a shift in gene expression and metabolic flux towards the biosynthesis of secondary metabolites and amino acids in reaction to cold stress treatment (HT2d-vs-GD2d) (**Figures 4C, D**). Genes participating in the biosynthesis of amino acids and flavonoids were down-regulated, causing a shift in both gene expression and metabolic flux toward the biosynthesis of secondary metabolites after 7 days of cold stress treatment (HT7d-vs-GD7d) (**Figure 4E**). Inclusively, these research findings suggest that numerous genes encoding core enzymes associated with the biosynthesis process of flavonoids and other crucial metabolites exhibited differentially expression. This implies that these genes could serve as valuable targets for GD cultivar, which appears to be sensitive to cold stress.

### Confirming gene expression through qPCR

**Figure 5:**
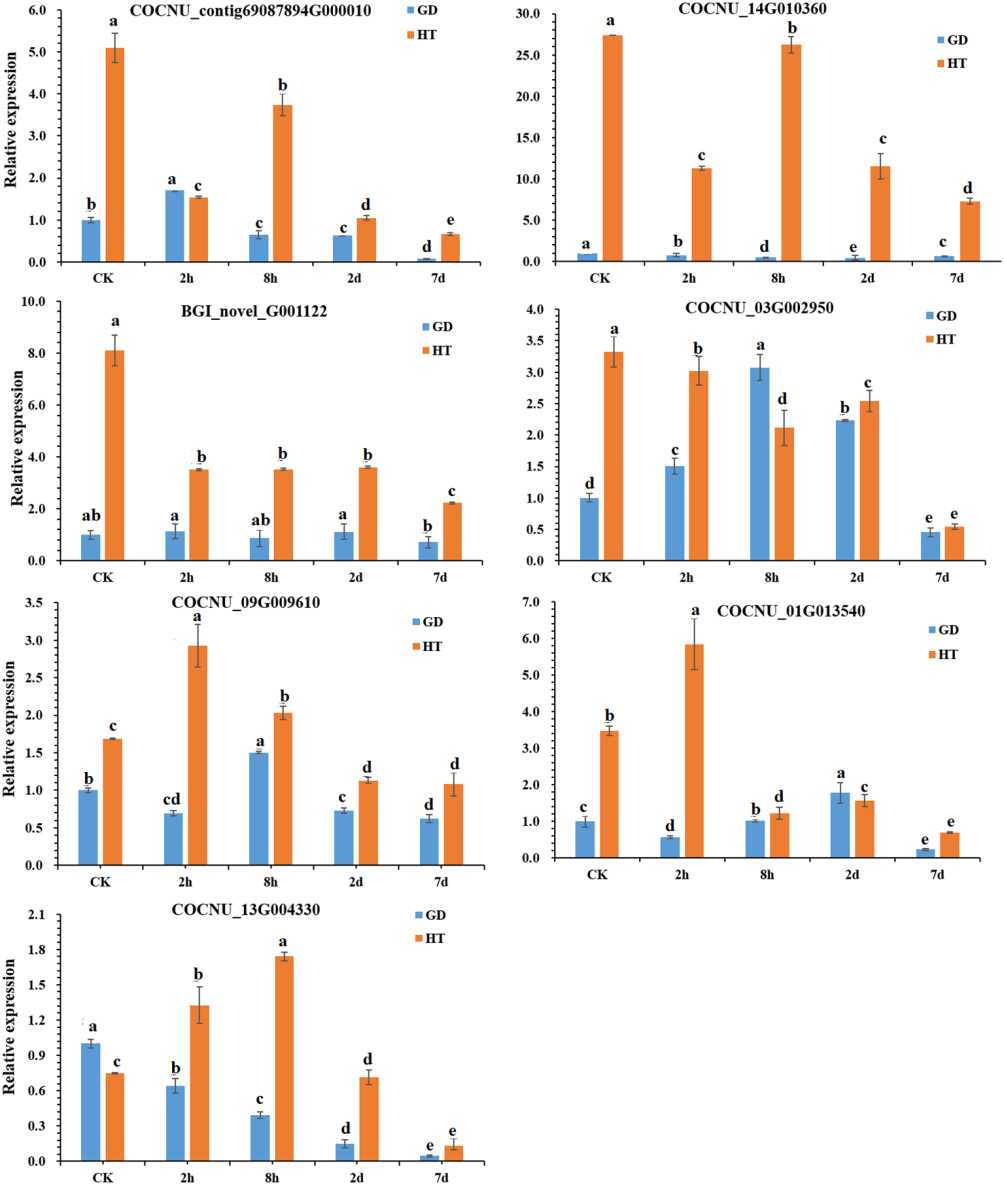
Expression levels of key genes in two coconut varieties under cold stress.Data represent the mean ± SD of three independent experiments. Different lowercase letters indicate significant differences according to the least significant difference test (LSD) at p < 0.05.

Using the transcriptomic and metabolomic data of two coconut varieties of HT and GD, we further analyzed the GO, KEGG and correlation of DEGs. In this analysis, we identified several important genes that are related to temperature stress. According to our transcriptomic and metabolomic data, we randomly selected (7) important genes (**Table 1**) for further validation through qPCR analysis. This allowed us to observe their expression levels under varying durations of temperature stress. In our gene expression data, it was observed that the genes COCNU_contig69087894G000010 (phosphoribosyl-ATP pyrophosphohdrolase), COCNU_14G010360 (flavonol synthase), BGI_novel_G001122 (phosphoglycerate kinase), COCNU_09G009610 (glutathione reductase (NADPH) genes were found upregulated in HT compared to GD across all time points (CK, 2h, 8h, 2d and 7d) under both low and high temperature. Intriguingly, the expression level of COCNU_03G002950 (glutamine synthetase) gene was specifically higher in GD compared to HT at the 8h time point (**Figure 5**).

**Table 1:** Annotation and expression of 7 genes in five comparative groups.

Gene ID	Annotation	log2(GD_CK_ /HT_CK_)	log2(GD_2h_ /HT_2h_)	log2(GD8h /HT8h)	log2(GD2d /HT2d)	log2(GD7d /HT7d)	KEGG
COCNU_14G01036	flavonol synthase	-3.85	-3.81	-4.50	-3.75	-4.98	ko00941 Flavonoid biosynthesis
COCNU_13G004330	flavonol synthase	-3.92	-2.66	-2.14	-1.88	-4.69	ko00941 Flavonoid biosynthesis
COCNU_09G009610	glutathione reductase (GR)	-1.07	-1.09	-1.46	-1.38	-1.08	ko00480 Glutathione metabolism
BGI_novel_G001122	phosphoglycerate kinase	-1.71	-2.67	3.01	-2.04	-2.95	ko01230 Biosynthesis of amino acids
COCNU_01G013540	indole-3-glycerol phosphate synthase	-0.63	-1.04	-1.06	-0.93	-0.90	ko01230 Biosynthesis of amino acids
COCNU_03G002950	glutamine synthetase	-1.76	-1.72	-1.64	-1.28	-0.93	ko00220 Arginine biosynthesis
COCNU_contig69087894G000010	phosphoribosyl-ATP	-4.25	-2.12	-3.76	-1.39	-1.21	ko01230 Biosynthesis of amino acids

In case of COCNU_01G013540 (indole-3-glycerol phosphate synthase), gene expression showed a bit higher in GD than HT at 2d, although this difference was non-significant. Moreover, the COCNU_13G004330 (flavonol synthase) gene exhibited a higher expression level in GD for CK compared to HT. However, when considering these three instances, levels of expression of all seven genes were consistently superior in HT than GD. This suggests that these genes are highly expressed under cold stress in HT variety.

**Figure 6:**
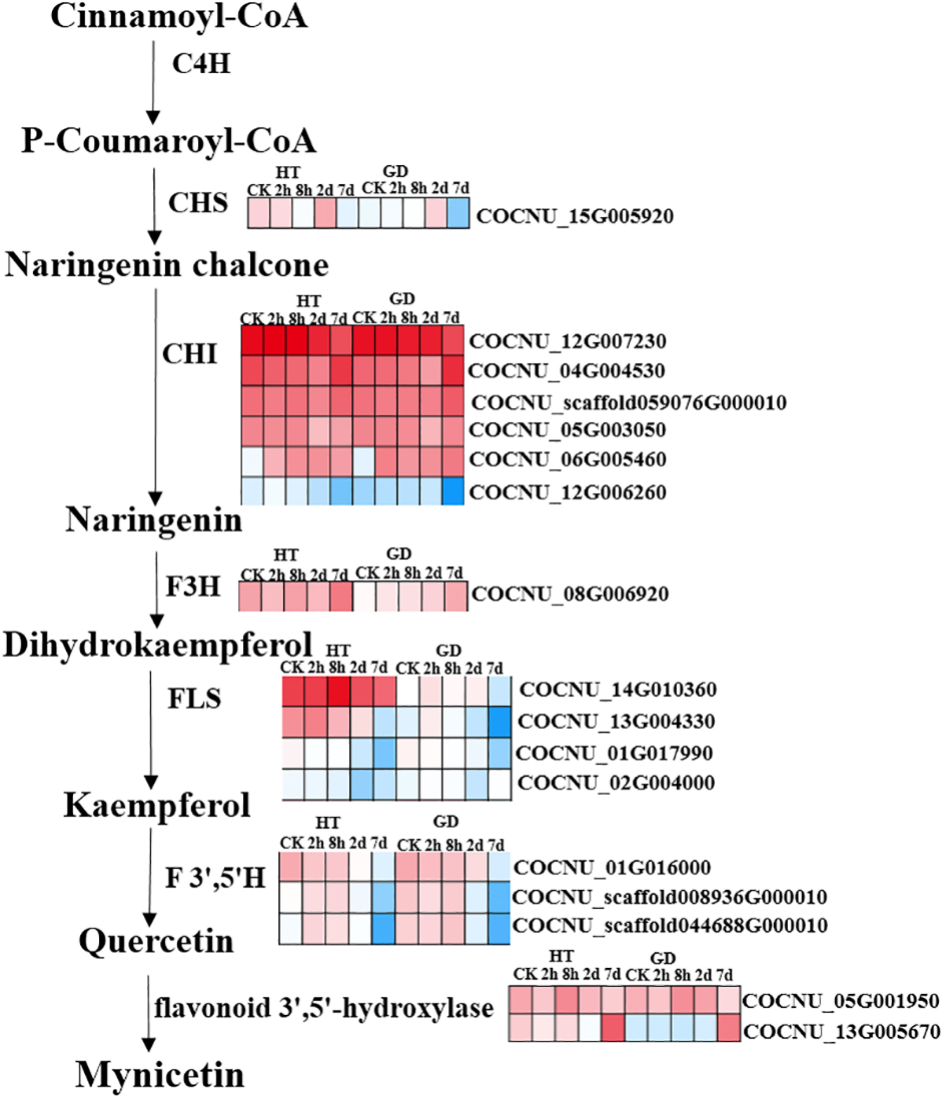
Different expressions of structural genes in flavonoid biosynthesis pathway. Color scale indicated FPKM value for genes. Red color represent relatively high levels of DEGs and blue color represent relatively low levels of DEGs.

In plants, the phenolic compounds like flavonoids, flavone, isoflavonoids etc. are commonly called as polyphenols are generated from the precursor like phenylalanine, tyrosine through the general phenylpropanoid pathway and this pathway is able to beget not less than 6000 phenolic compounds. However, phenolic compounds like flavonoids played a crucial role to develop defense system in plants under stress condition and flavonoids synthesis is linked to the phenylpropanoid biosynthesis pathway (map 00941) (Hichri et al., 2011). In this pathway PAL, cinnamate 4-hydroxylase, and 4-coumarate-CoA ligase successively catalyze the synthesis of the substrate 4-coumaroyl-CoA, a crucial component for flavonoid biosynthesis. Coumaroyl CoA undergoes conversion by CHS into chalcone, and this chalcone is further transformed by CHI into naringenin (Wen et al., 2020). The integrated results from metabolomics and transcriptomics indicate the significance of flavonoids in the plant’s reaction to cold stress. Consequently, we proceeded to delve further into the biosynthetic pathway of flavonoids in coconuts (**Figure 6**). As the duration of exposure to cold stress increased, we observed a decrease in the transcription levels of COCNU_14G010360 and COCNU_13G004330 in both varieties. This finding aligns with the results obtained from subsequent qPCR analyses. Remarkably, the transcription levels of COCNU_14G010360 and COCNU_13G004330 in Hainan tall coconut were found to be significantly higher than those in GD coconut (**Figures 5**, **6**).

## Discussion

### Hainan tall coconut exhibits superior cold tolerance compared to green dwarf coconut

The coconut is among the most extensively distributed and cultivated tropical fruit tree species. However, the impact of low temperatures has constrained its global spread and production. Exposure to cold stress impacts the seedling growth of various plants, including coconut, by causing membrane disruption, gene and protein dysfunction, alterations in carbohydrate, protein, and lipid metabolism, fluid leakage, necrosis, chlorosis, and other molecular, physiological, and morphological changes. These changes hinder the plant’s ability to respond promptly to unfavorable environmental conditions, affecting proper growth and development (Ritonga and Chen, 2020; Hassan et al., 2021; Lu et al., 2023). The exposure to low-temperature treatment markedly decreased growth parameters of coconut seedlings, including plant height and dry weight. Unraveling the molecular mechanisms underpinning the reaction of coconut varieties at the seedling stage to cold stress can serve as a foundation for enhancing their resistance to low temperatures (Lu et al., 2023). Past studies indicate a positive correlation between osmoprotectants and the tolerance of plants to cold stress (Fu et al., 2020; He et al., 2021). Plants adapt to cold stress by accumulating osmotic regulators, including soluble sugars (SS), proline (Pro), and soluble proteins (SP) (Raza et al., 2022). The amino acid proline (Pro) tends to accumulate in response to low temperature stress. This accumulation has been suggested to contribute to enhanced tolerance to low temperature stress in rice, as indicated by the balanced relationship between Pro accumulation and stress tolerance (Verbruggen and Hermans, 2008). Soluble protein (SP) accumulation is another response observed in plants under low temperature stress. Moreover, it is recognized for its role as an osmoprotectant, providing protection against dehydration damage (Guo et al., 2022). In our study, we observed an increase in the levels of proline and soluble protein in coconut seedlings under low temperature stress (**Figures 1B, C**). Under cold stress, plants typically show a decrease in MDA content along with an increase in the activities of SOD and POD (Gill and Tuteja, 2010). Du et al. (2020) also found higher SOD activity was in a chilling-tolerant rice variety compared to a chilling-susceptible variety. During our research, we also noted that following 7 days of cold stress treatment, GD seedlings showed significant frost spots on their leaves, while HT seedlings showed no significant difference compared to CK (**Figure 1A**). Cold stress simultaneously increased SOD and POD enzyme activity in HT and GD leaves, but the degree of increase was much higher in HT leaves compared to the GD leaves (**Figures 1E, G**). Proline has important roles during stress signal transduction, and it also serves as an antioxidant. The increase in proline levels under various stresses has been reported in previous studies (Liu et al., 2018). In the course of this investigation, as the duration of cold stress lengthened, the levels of SP and PRO increased, and the magnitude of the increase in HT was higher than that in GD. Additionally, the activity of glutathione oxidase decreased, indicating that HT exhibited a higher resistance to cold stress compared to GD coconuts (**Figures 1B–D**). These findings align with the previous results of Yang et al. (2020), who observed that HT displays greater cold tolerance compared to the aromatic coconut variety, GD.

Unraveling the mechanism by which coconuts respond to cold stress can contribute to enhancing their resistance to low temperatures. In this investigation, we scrutinized two coconut varieties with marked differences in their tolerance to cold stress. Aromatic coconut, characterized as a type of GD coconut, was included in the study. Our analysis involved identifying the metabolic and transcriptional profiles of coconut varieties exhibiting varying degrees of tolerance to cold stress, aiming to gain a more profound understanding of the mechanisms underlying their variations in tolerance. By conducting a combined analysis of the transcriptome and metabolome, we observed a significant reduction in stress- adaptive genes and metabolites in the low-temperature-sensitive coconut compared to the low-temperature-resistant coconut.

### Amino acid pathway metabolites may contribute to cold tolerance in coconut

Under abiotic stress conditions, specific high-abundance amino acids like proline, arginine, asparagine, glutamine, and GABA are produced, serving as compatible osmolytes, building blocks for secondary metabolites, or reservoirs of organic nitrogen (Hildebrandt, 2018). Compared with the transcriptional and metabolic profiles two varieties while undergoing cold acclimatization, it was attained that amino acid biosynthesis pathway might be responsible for the differences in the cold resistance. Free amino acids can act as osmolytes in plant serve a pivotal purpose in developmental and metabolic dynamics in plants by regulating gene expression and redox homeostasis (Jiang et al., 2022). Cold resistance inNicotiana tabacum exhibited a positive correlation with elevated levels of proline and frees amino acids (Song et al., 2024). The accrual of amino acids is a commonly observed phenomenon in numerous plant species under conditions of cold stress (Zhang et al., 2019; Cheng et al., 2023). Research indicated that cold-tolerant tea varieties exhibit a substantially greater accumulation of amino acids compared to cold-sensitive tea varieties during cold acclimation (Wang et al., 2022). In the course of this study, metabolome analysis uncovered that the associated metabolites were predominantly concentrated in pathways related to flavonoids, carbohydrate, amino acid, lipid, and nucleotide metabolism. This enrichment was observed under conditions of cold stress. In the amino acid metabolism pathways, L-(+)-Arginine, D-(-)-Glutamine, L-Pyroglutamic acid and aspartic acid, is more enriched under cold stress. Specifically, in HT, the abundance of arginine increased under cold stress, while it was not detected in GD. We conducted a comparative analysis of the transcriptional and metabolic profiles of Hainan Tall and Green Dwarf varieties during cold acclimation. Our findings suggest that the biosynthesis of amino acids may be the pathway accountable for the observed differences in the cold resistance of coconut cultivars. This discovery is consistent with the results in tea trees (Cheng et al., 2023).

In the current investigation, we observed down-regulation of genes associated with amino acid biosynthesis, specifically those encoding the phosphoglycerate kinase PGK (BGI_novel_G001122), indole-3-glycerol phosphate synthase IGPS (COCNU_01G013540), glutamine synthetase GS (COCNU_03G002950), phosphoribosyl-ATP, pyrophosphohydrolase/phosphoribosyl-AMP cyclohydrolase/histidinol dehydrogenase (COCNU_contig69087894G000010), during cold stress in the low-temperature-sensitive coconut variety GD (**Table 1**). This expression pattern differed from the low-temperature-insensitive coconut variety HT, indicating a potential role of these genes in the observed cold sensitivity. The biosynthesis of amino acids gradually decreased with the duration of cold stress treatment, indicating a mechanism that might be contributing to its decreased tolerance. Among them, glutamine synthetase GS (COCNU_03G002950) is involved in arginine biosynthesis.

### Flavonol metabolism pathway metabolites may contribute to cold tolerance in coconut

Temperature serves as the primary environmental factor regulating plant secondary metabolites, particularly flavonoids (Korn et al., 2008; Fini et al., 2011; Zhang et al., 2022). The stress resulting from a temperature decrease (4-10°C) promotes the accumulation of flavonoids. Exposure to cold conditions enhances the flavonoid content in leaves, leading to enhanced tolerance to UV-B radiation (Bilger et al., 2007). Moreover, plants produce elevated levels of ROS in response to stress, and quercetin exhibits the highest capacity for scavenging radicals (Baek et al., 2015). The levels of certain flavonoids show a notable correlation with plant freezing tolerance following cold acclimation (Schulz et al., 2015). Studies involving various biosynthetic mutants have unveiled the functional role of flavonoids in Arabidopsis freezing tolerance and cold acclimation (Schulz et al., 2021). Research has demonstrated that tea plants produce elevated levels of flavonoids, including anthocyanins, quercetin, and kaempferol, in response to cold stress, serving as a mechanism to resist oxidative damage (Zheng et al., 2016). Recent research has indicated that cold stress triggers the expression of structural genes within the phenylpropanoid pathway, such as 4-coumarin-CoA ligase (4CL), and chalcone synthetase (CHS) (Gouot et al., 2019). As reported, the expression of genes involved in flavonoid synthesis, such as CHS, PAL, FLS1, and F3H showed a significant up-regulation (Zhang et al., 2015; Petridis et al., 2016). The impact of cold on the accumulation of anthocyanins in grape skin was investigated and it was found that overnight at 10-11°C accelerated the accumulation anthocyanins. Molecular analysis revealed an increase in the transcription of four key genes (CHS, F3H, MYBA1, and UFGT) in the anthocyanins biosynthesis pathway (Gaiotti et al., 2018). Consequently, the biosynthesis of flavonoids was promoted (Schulz et al., 2016). Currently, it has been observed that low temperatures can markedly stimulate the accumulation of flavonoids in ginkgo leaves (Guo et al., 2020), Arabidopsis (Schulz et al., 2015). Specifically, the levels of kaempferol and quercetin exhibit a significant increase under conditions of cold stress.

The expression of pivotal genes in the phenylpropanoid pathway, the biosynthetic route of flavonoids, and antioxidant system showed significant upregulation at the time of pyroxsulam treatment in pyroxsulam-resistant highland barley (Weng et al., 2022). During overwintering cultivation, an accumulation of flavonols was observed, leading to the analysis of synthesis genes. The increased expression of BcCHS, BcF3H, and BcFLS1 pointed to an enhanced synthesis of flavonols (Chen et al., 2023). Coconut under K+ deficiency, flavonoid, phenolic levels, antioxidant enzymes activities and other secondary metabolites have been changed significantly (Lu et al., 2023). Pathway analysis indicated that “flavonoid biosynthesis,” “anthocyanin biosynthesis,” “flavone and flavonol biosynthesis,” and “plant hormone signal transduction” may play a vital role in the response of Dendrobium huoshanense to cold stress (Wu et al., 2023). Our Pathway analysis also found that “flavonoid biosynthesis,” and “flavone and flavonol biosynthesis” play a major role in coconut low-temperature stress. According to KEGG enrichment analysis, the most DEGs-enriched KEGG pathways in our study included “plant hormone signal transduction”, “MAPK signaling pathway-plant”, “biosynthesis of amino acids”, “carbon metabolism”, “starch and sucrose metabolism”, “glycolysis/gluconeogenesis”, “phenylpropanoid biosynthesis”, “steroid biosynthesis”, “plant-pathogen interaction” and “amino sugar and nucleotide sugar metabolism” (**Figure 4**). Similar results were found in the cold-stress response of *Brassica napus* (Raza et al., 2021, Song et al., 2023). In this investigation, we found that biosynthesis of flavonoids-related genes encoding the flavonol synthase FLS (COCNU_14G010360 and COCNU_13G004330) were significantly down-regulated during cold stress in the low-temperature-sensitive coconut variety GD compared with the low-temperature-insensitive coconut variety HT (**Table 1**; **Figure 5**). The flavonoid content gradually decreased with the duration of cold stress treatment that could be a factor contributing to its lower tolerance. The outcomes offer an initial insight into leveraging metabolomics for the cultivation of cold stress-tolerant coconut varieties.

### Cold-induced changes in antioxidative defense mechanisms of coconut

Within plant cells, the cytoplasm, chloroplasts, and mitochondria produces ROS such as superoxide radicals (O_2_^-^), hydrogen peroxide (H_2_O_2_) and hydrogen radicals (OH^-^), causing damage to lipid, protein, cell membrane and cell wall (Gupta and Huang, 2014). Plant can be alleviated or minimized the ROS formation by some non-enzyme antioxidants and detoxifying enzymes which are SOD, ascorbate APX and glutathione reductase by linking to different complexes of electron transport system (ETS) (Smith et al., 2009). Cellular responses can be triggered by various environmental factors, such as cold (Li et al., 2019), heat (Zhao et al., 2018), and drought (Zheng et al., 2020). The impact of cold stress manifests in the accumulation of ROS within cells, culminating in the inactivation of metabolic enzymes. Additionally, cell membranes, proteins, and nucleic acids become susceptible targets, causing cellular damage in the form of oxidative stress (Gill and Tuteja, 2010). In response to ROS-induced stress, plants deploy enzymatic antioxidants like superoxide dismutase (SOD), ascorbate peroxidase (APX), catalase (CAT), guiacol peroxidase (GPX), alongside non-enzymatic antioxidants such as glutathione and carotenoids to alleviate the negative consequences (Heidarvand and Maali-Amiri, 2013). Also, the germination process of maize seeds under chilling stress was found to be associated with a marked increase in the activities of antioxidant enzymes (Cao et al., 2019). Peroxidases are a kind of antioxidant enzymes, which can remove ROS in plant cell (Buer et al., 2016). In plants, the function of glutathione is particularly important as it participates in regulating life activities such as plant growth, development, and stress response (Li Z. et al., 2023; Liu et al., 2023). Glutathione, serving as an antioxidant, helps the body rid itself of free radicals and peroxides, thereby upholding the relative stability of the organism. Regulating redox-sensitive signal transduction in plant tissues, glutathione metabolism is crucial for maintaining their antioxidant properties (Cnubben et al., 2001). Compared with the low-temperature-insensitive coconut variety HT, glutathione metabolism-associated genes encoding glutathione reductase GR (COCNU_09G009610), were down-regulated in the low-temperature-sensitive coconut variety GD (**Table 1**; **Figure 5**). The down-regulation of genes related to the antioxidant system is evidenced in the results, causing a decline in antioxidants. This, in turn, results in the accumulation of reactive ROS in the sensitive coconut variety (GD). This may be one of the reasons for its low temperature intolerance.

## Conclusions

In the low-temperature stress-sensitive coconut (GD), a marked decrease in the relative expression of antioxidant-related genes, compared to the low-temperature stress-resistant coconut (HT), contributed to a decline in antioxidants. Numerous metabolites, such as amino acids and flavonoids have the potential to act protectively in the face of cold stress. Considering all the evidence, it can be inferred that coconut varieties exposure to cold stress played a pivotal role to changes in antioxidants, flavonoids and amino acids contents. It can be inferred from this observation that flavonoids, antioxidant enzyme and amino acids genes were decreased under cold stress in GD variety. Our study unveils new understandings of the molecular mechanisms involved in cold stress tolerance in coconut. This paves the way for the initial stages in the development of cold-tolerant coconut varieties and other crops, utilizing gene editing or traditional breeding approaches.

## Data availability statement

The original contributions presented in the study are included in the article/**Supplementary Material**. Further inquiries can be directed to the corresponding author.

## Author contributions

JL: Writing – review & editing, Writing – original draft. FW: Writing – original draft. MS: Writing – review & editing. XJS: Writing – review & editing, Software. LZ: Writing – review & editing. XL: Writing – review & editing. XWS: Writing – review & editing. SC: Writing – review & editing. YW: Writing – review & editing. LL: Writing – review & editing. SG: Writing – review & editing. AI: Writing – review & editing. YY: Writing – review & editing.

## Supplementary material

Supplementary Figure 1 KEGG annotation ring of DEMs.

Supplementary Figure 2 Metabolite classification of DEMs.

Supplementary Figure 3 The number of DEGs between the five cold treat periods.

Supplementary Figure 4 Venn diagram of enrichment pathways from different comparisons.
