# Supplementary figures and images for: Integrated transcriptomic and metabolomic data reveal the cold stress responses molecular mechanisms of two coconut varieties
Source: Front Plant Sci. 2024 Apr 16;15:1353352. doi: 10.3389/fpls.2024.1353352 (PMC11058665; doi:10.3389/fpls.2024.1353352)

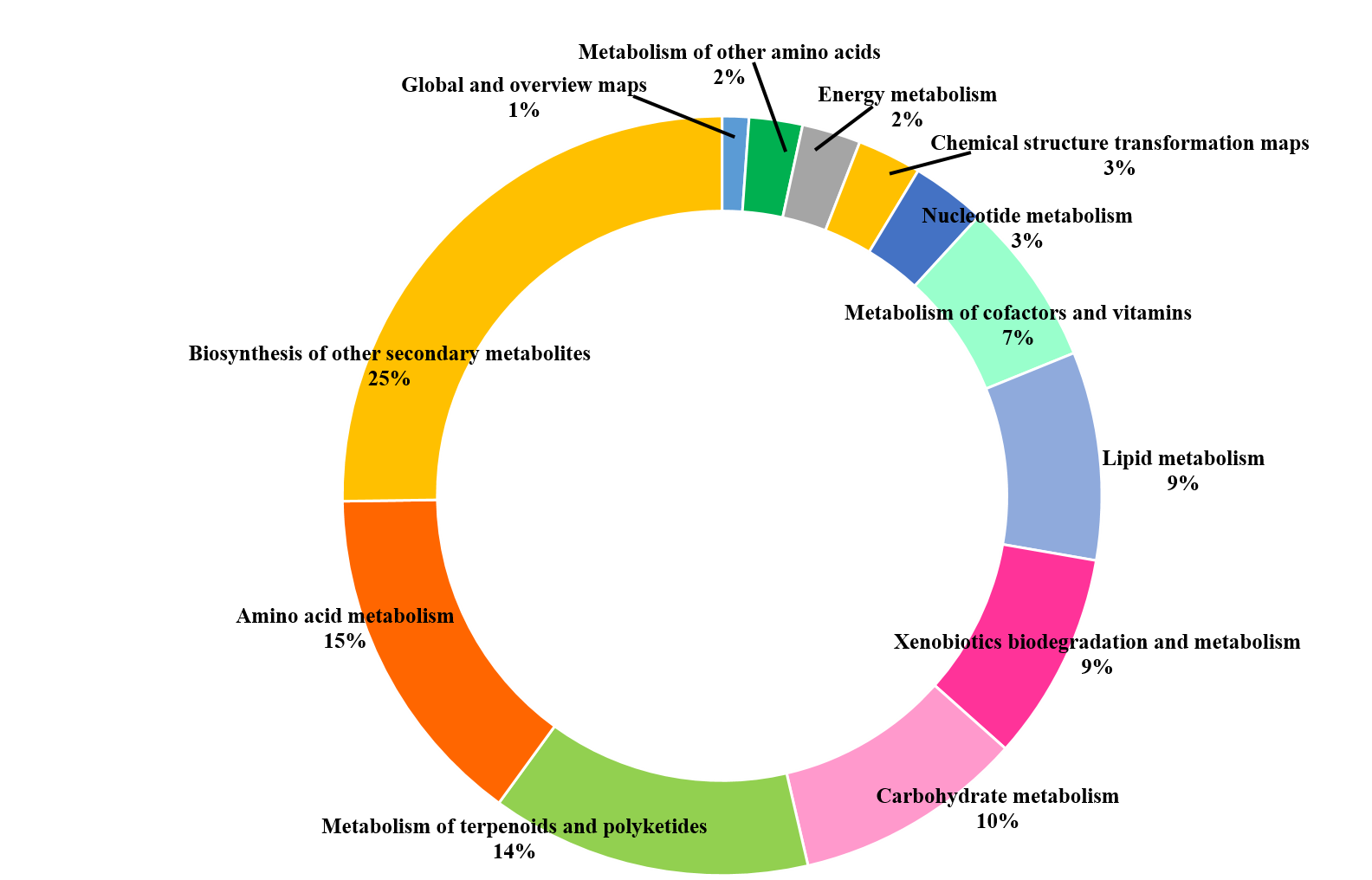

Supplement: Supplementary Figure 1 — KEGG annotation ring of DEMs. [file Image_1.tif]

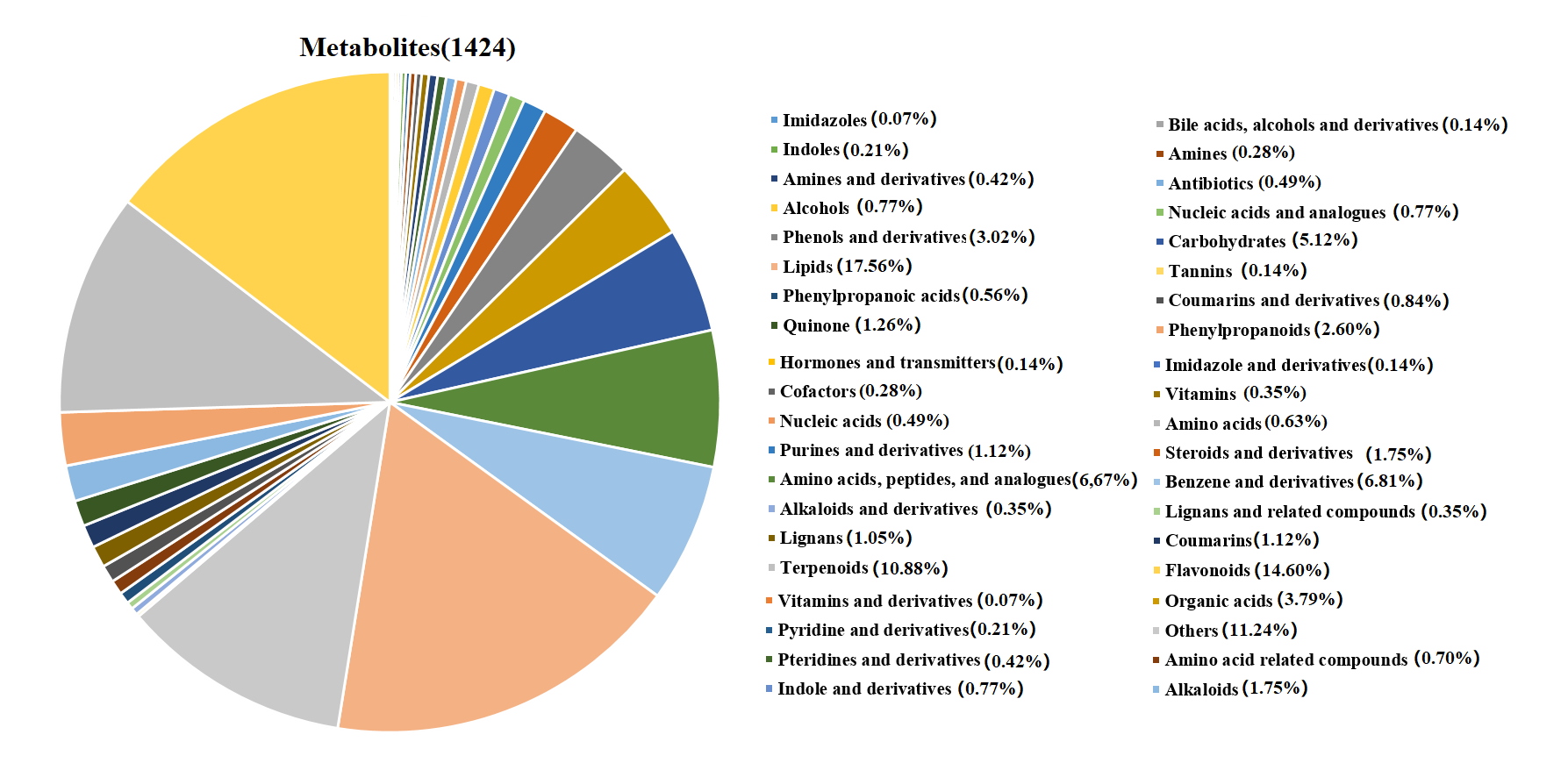

Supplement: Supplementary Figure 2 — Metabolite classification of DEMs. [file Image_2.tif]

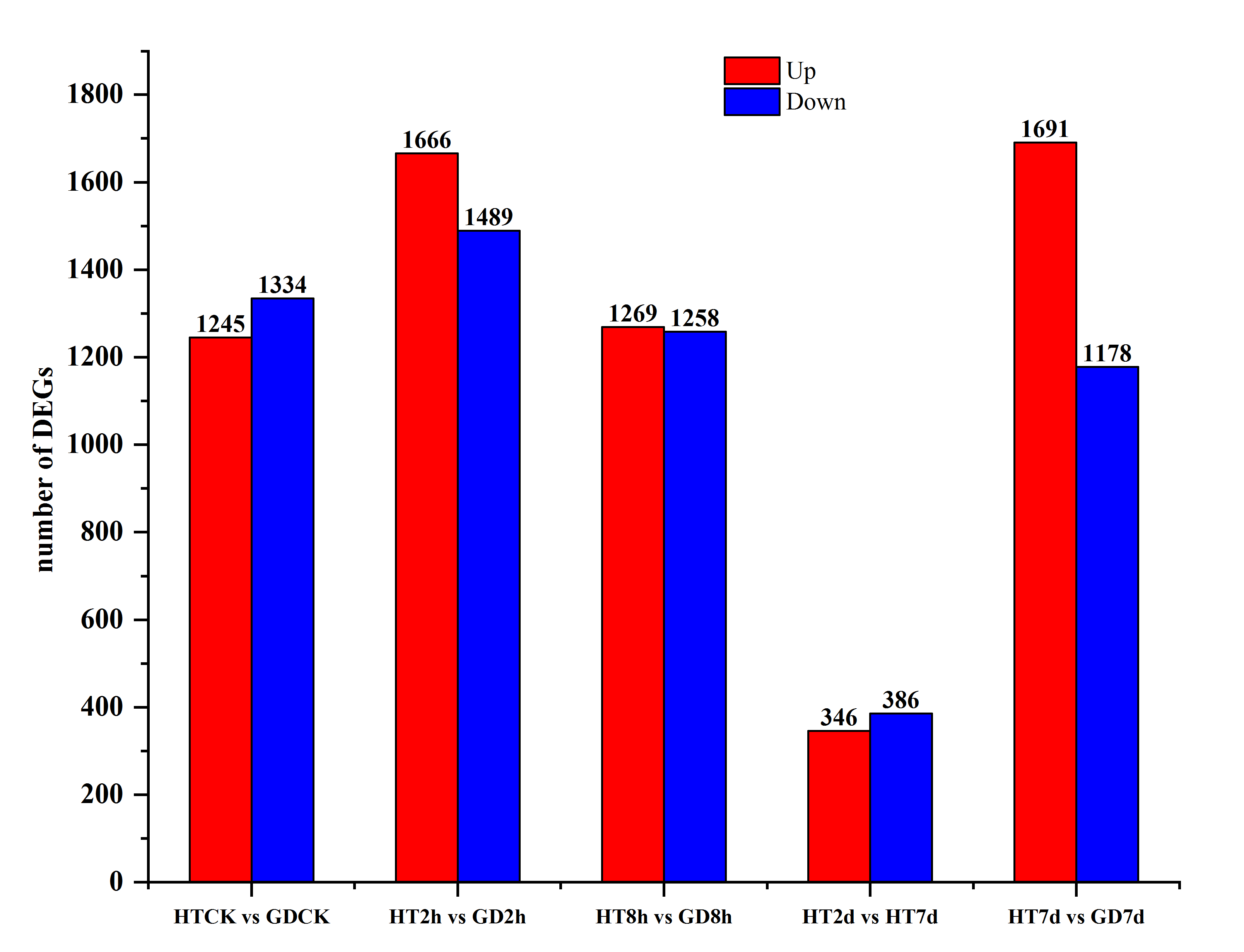

Supplement: Supplementary Figure 3 — The number of DEGs between the five cold treat periods. [file Image_3.tif]

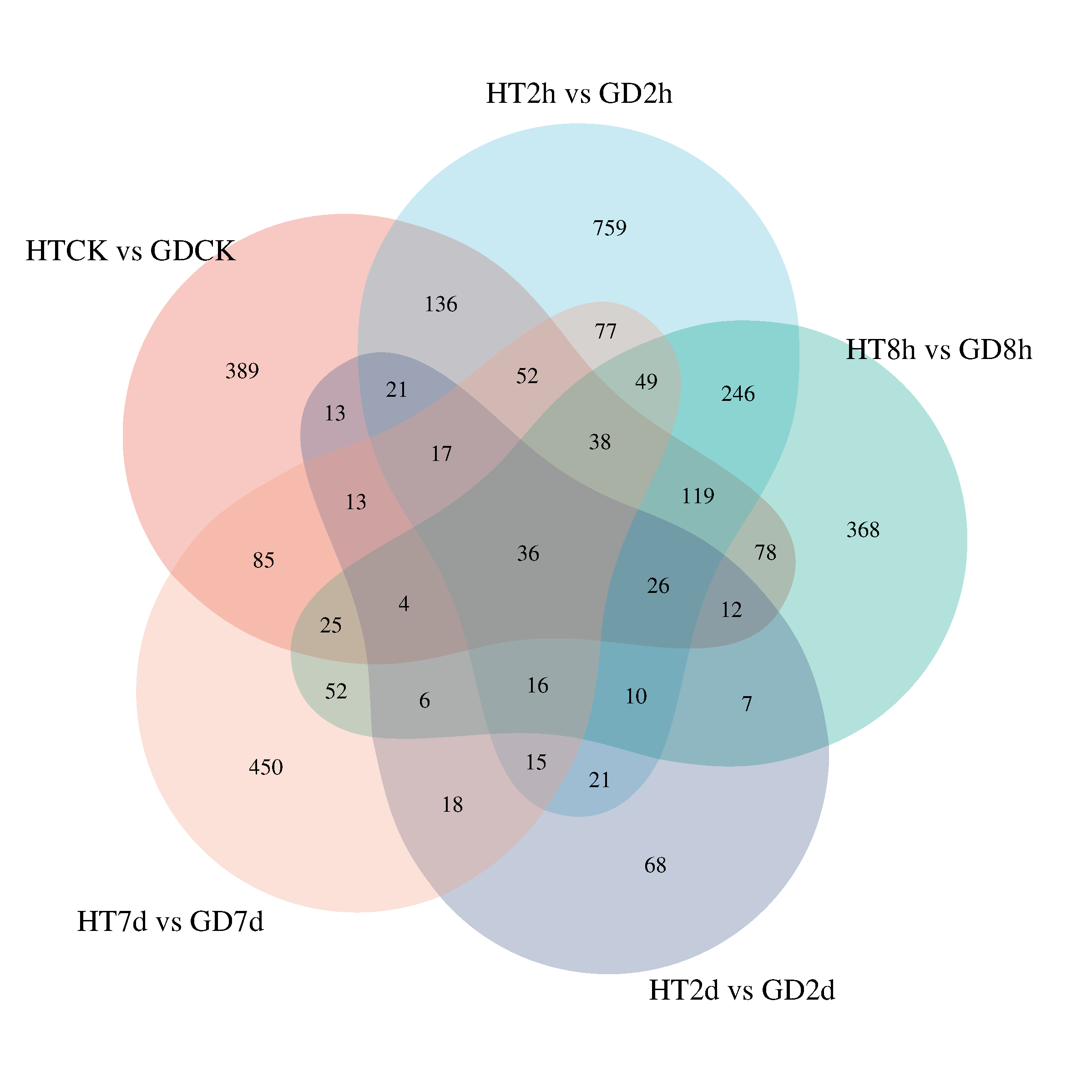

Supplement: Supplementary Figure 4 — Venn diagram of enrichment pathways from different comparisons. [file Image_4.tiff]
